# A Novel Nonsense Mutation (c.414G>A; p.Trp138*) in *CLDN14* Causes Hearing Loss in Yemeni Families: A Case Report

**DOI:** 10.3389/fgene.2019.01087

**Published:** 2019-11-08

**Authors:** Walaa Kamal Eldin Mohamed, Mona Mahfood, Abdullah Al Mutery, Sallam Hasan Abdallah, Abdelaziz Tlili

**Affiliations:** ^1^Department of Applied Biology, College of Sciences, University of Sharjah, Sharjah, United Arab Emirates; ^2^Departament de Genètica i de Microbiologia, Facultat de Biociències, Universitat Autònoma de Barcelona, Barcelona, Spain; ^3^Human Genetics & Stem Cells Research Group, Research Institute of Sciences & Engineering, University of Sharjah, Sharjah, United Arab Emirates

**Keywords:** *CLDN14* gene, clinical exome sequencing, nonsense variant, non-syndromic hearing loss, founder effect

## Abstract

Non-syndromic hearing loss (NSHL) is a hereditary disorder that affects many populations. Many genes are involved in NSHL and the mutational load of these genes often differs among ethnic groups. Claudin-14 (*CLDN14*), a tight junction protein, is known to be associated with NSHL in many populations. In this study, we aimed to identify the responsible variants in 3 different Yemeni families affected with NSHL. Firstly, clinical exome sequencing (CES) performed for 3 affected patients from these different families identified a new nonsense variant (c.414G > A) in *CLDN14*. This variant was then confirmed by Sanger sequencing and PCR-RFLP. Subsequently, four microsatellite markers were used to genotype these families, which revealed a founder effect for this variant. Overall, this study illustrates the implication of the *CLDN14* gene in the Yemeni population with NSHL and identifies a new founder variant.

## Background

Hearing loss (HL) is a relatively common congenital disorder; its prevalence in newborns is 1 in 1,000 live births (MORTON, 1991). Hereditary HL can be associated with other symptoms but in the majority of cases (70%) it is non-syndromic (Najmabadi and Kahrizi, 2014). Non-syndromic hearing loss (NSHL) can be classified by its mode of inheritance as; autosomal dominant (∼15–20%), autosomal recessive (∼80%), X-linked, Y-linked or mitochondrial (together ∼2%) (; Hone and Smith, 2002).

Autosomal recessive non-syndromic hearing loss (ARNSHL) is extremely genetically heterogeneous with 72 genes and 108 loci associated with this type of HL (https://hereditaryhearingloss.org). Next Generation Sequencing (NGS) is an important approach for screening diseases with high heterogeneity, such as hearing loss, as it can quickly characterize hundreds of genes and a large number of common variants (http://hearing.harvard.edu/db/genelist.htm) in timely and cost effective manner as opposed to Sanger sequencing (Gürtler et al., 2008; Gao et al., 2015).

Variants in gap and tight junctions are known to be associated with ARNSHL (Martínez et al., 2009; Kim et al., 2014). Connexins are important for the formation of gap junctions between adjacent cells, where direct intercellular communication *via* diffusion of ions and metabolites can occur (Goodenough and Paul, 2003); members of the connexin family have an important role in ARNSHL. In fact, many reports claim that approximately 50% of autosomal recessive HL cases are caused by either homozygous or compound heterozygous variants in the gap junction beta 2 (*GJB2*) gene (Zheng et al., 2015), making the *GJB2* gene the most frequent gene implicated in ARNSHL. Therefore, it is common to pre-screen NSHL individuals for *GJB2* variants, to determine whether NGS techniques are required. In the inner ear, tight junctions are involved to maintain the difference in concentration between the endolymph and the perilymph. The *CLDN14* gene, encoding a tight junction protein, serves as potassium-restrictive barrier in the Corti organ (Ben-Yosef et al., 2003). Since the identification of *CLDN14* variants in families with ARNSHL (Wilcox et al., 2001), several genetic and functional studies have been performed. These studies confirmed the implication of the *CLDN14* gene in hearing loss (Lu et al., 2018).

In this study, we performed Clinical Exome Sequencing (CES) and identified a new nonsense variant in three different Yemeni families. Haplotype analysis of the *CLDN14* gene region, using four microsatellite markers, showed that this pathogenic DNA variant has a founder effect.

## Case Presentation

**Figure 1:**
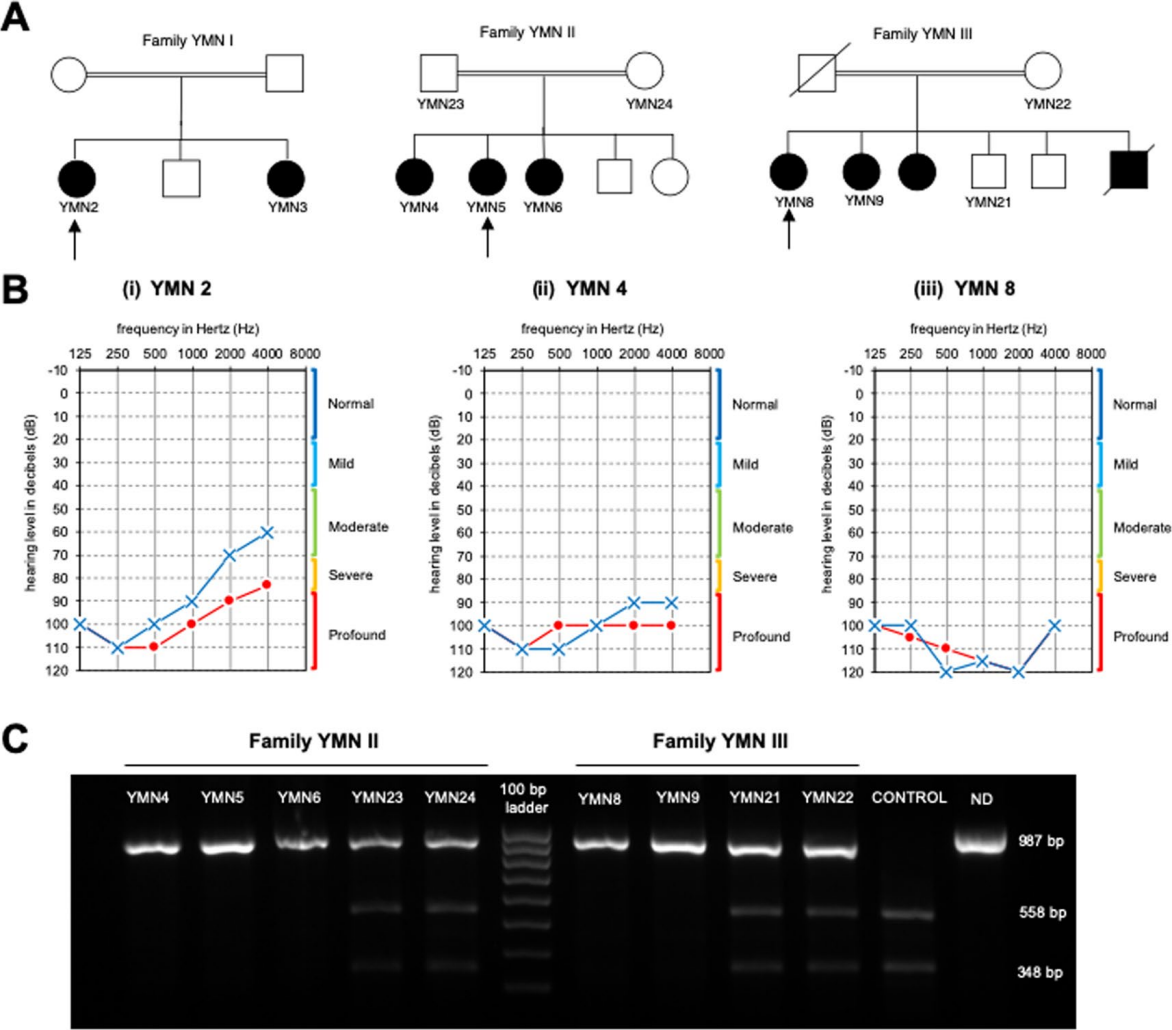
Pedigrees of the affected families, Audiograms and PCR-RFLP. **(A)** Pedigrees of three affected families with non-syndromic hearing loss. Arrows denote the probands. **(B)** Audiograms of the affected probands from the 3 Yemeni families: (o) indicates air conduction for right ear, (x) indicates air conduction for left ear. **(C)** Results of the PCR-RFLP analysis in YMNII and YMNIII affected families. The 987 PCR product digested with the AvaII restriction enzyme. The wildtype allele is cleaved into three fragments 558, 348, and 81 bp, whereas the c.414G>A mutant allele is cleaved into two fragments 906 and 81 bp in length. ND, undigested PCR product.

In this study, we investigated three consanguineous Yemeni families diagnosed with ARNSHL (**Figure 1A**). The proband YMN2 of the family YMN-I is a 25 year old affected female. Her audiogram showed an average pure tone air conduction (PTA) varying from 60–80 for her left and right ears (**Figure 1**) and the otoscopy of both ears was normal. The proband’s younger sister (YMN3) is 23 years old and showed an average PTA of 70–80; her otoscopy was also normal (**Supplementary Figure 1**). The proband’s younger brother and parents are phenotypically normal. The YMN-II family consists of three affected female siblings, YMN4 is 29 year old, YMN5 is 14 year old and YMN6 is 21 year old with PTA of 90–100, 70–80 and 80 for left and right ears respectively, their otoscopy was also normal. Moreover, the proband’s third sister, brother and parents are phenotypically normal (**Figure 1** and **Supplementary Figure 1**). The last family YMN-III consisted of two affected female siblings YMN8 and YMN9 (25 and 29 years old respectively) with PTA of 100, 90–100 for left and right ears (**Figure 1** and **Supplementary Figure 1**), the otoscopy result for both affected females was normal. The proband in this family has an elder affected sister (sample could not be collected), deceased affected brother, two phenotypically normal brothers and parents. In summary, the analysis of the audiograms of the three families (**Supplementary Table 1**), showed a sloppy audiogram shape in all the affected individuals.

## Materials and Methods

### Sample Collection

Collection of samples was done in collaboration with Al-Amal association of deaf and mutism in Hadramout coast (Mukallah, Yemen). Consent forms were filled and signed by all participants. Saliva samples were collected from the participants using Oragene-DNA (OG-500) Kit (DNA Genotek, CANADA), and only letters and numbers were used to label DNA samples to protect the participant’s privacy. Genomic DNA was extracted using Prep IT L2P (DNA Genotek, CANADA) following the manufacturer’s protocol. The experimental procedures were approved by the Ethics Committee from the University of Sharjah (Sharjah, UAE) and by the General Directorate of the Office of the Ministry of Health and population-Hadramout Coast (Mukallah, Yemen).

### Clinical Exome Sequencing and Bioinformatics Analysis

Clinical exome sequencing and standard data analysis was performed for the affected individuals YMN3, YMN5, and YMN8. The genomic DNA was sheared and used to perform exome capture using oligonucleotide probes (SureSelect V5+UTRs) following the manufacturer’s provided protocols. The exonic region was later enriched by hybridizing capture probes. The captured fragments were then adapted to produce libraries that were sequenced on the Illumina HiSeq 2500/4000 system (Illumina, San Diego, CA, USA) to generate paired end 2X 100bp sequence reads to produce 100x mean coverage. Only reads that were generated from high quality sequences were analyzed after quality control for variant calling and annotation, and later were aligned to the human reference genome build GRCh37/hg19 using BWA-0.7.12. PCR duplicates were then removed using Picard-1.140 and variants were called using Genome Analysis Toolkit (GATK) v2.3-9. Known variants were annotated with in-house Variation and Mutation Annotation Toolkit 2.3.4 (VariMAT). The remaining variants were then further filtered by frequency (i.e. < 0.01%) in dbSNP (https://www.ncbi.nlm.nih.gov/projects/SNP/) or ExAC Browser (http://exac.broadinstitute.org/). Finally, to predict the functional impact of the candidate variants the following bioinformatics tools were used: Variant Effect Predictor (VEP) (http://grch37.ensembl.org/Homo_sapiens/Tools/VEP) and VarSome (https://varsome.com/).

### Sanger Sequencing

Sanger sequencing was performed for all members of the investigated families to confirm the co-segregation of the c.414G>A variant with the disease phenotype. In order to amplify the exonic region corresponding to this variant, we designed the following primers: CLDN14-F: ACCACCATCCTGCCGCACTG and CLDN14-R: TGTTTGCAGTGGTCGTGGTG. Polymerase Chain Reaction (PCR) was then carried out at an annealing temperature of 55°C generating amplicons of 550 bp in length. The amplified products were then purified using ExoSapIT clean up reagent (Affymetrix, Fisher Scientific, Göteborg—Sweden). Cycle sequencing was performed using Big dye terminator V3.1 cycle sequencing kit (Applied Biosystems, USA). The products were later purified using Dye EX 2.0 spin kit (Qiagen, Germany) and injected in the 3500 Genetic Analyzer (Applied Biosystems, Thermo Fisher Scientific, USA). The sequences were aligned with the published sequence of the *CLDN14* gene (NG_011777.1; NP_001139549.1).

### Variant Screening Using PCR-RFLP

The AvaII restriction enzyme was used to screen the c.414G>A nucleotide transition in the affected families and healthy controls. The primers’ sequences were: CATTTCCTTTCTCTCCCTGCT and GACATTTCCTCGCATTCACA. The PCR product size was of 987 bp. The digestion of PCR products was performed according to the manufacturer’s instructions (New England Biolabs, USA) followed by separation on 2% agarose gel at 80 V for 1 h and half.

### Genotyping

**Figure 3:**
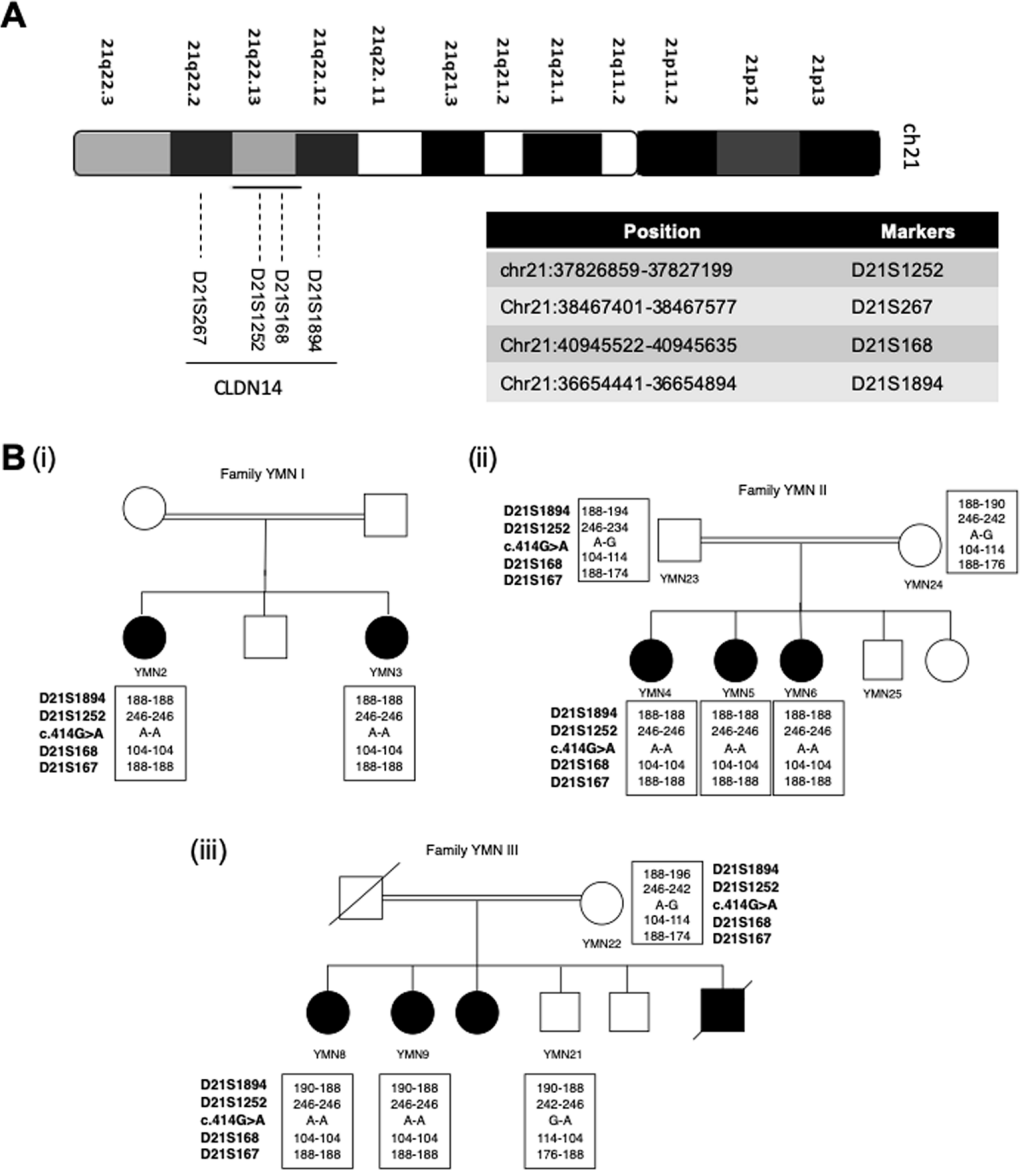
Founder effect of the c.414G>A variant. **(A)** Chromosomal location of the *CLDN14* gene and the 4 STR markers on chromosome 21 used to study the c.414G > A founder effect **(B)** Haplotype analysis in the 3 affected families showing the segregation of a common haplotype with the c.414G>A variant and confirming its founder effect.

Four microsatellite markers (D21S1252, D21S168, D21S267, and D21S1894), flanking the *CLDN14* gene chromosomal region were chosen (**Figure 3A**). The sequences of these microsatellite markers are described in **Supplementary Table 2**. For each marker, the forward primer was labeled with a specific fluorescence and the PCR products were analyzed using the Genetic Analyzer 3500 (Applied Biosystems, Thermo Fisher Scientific, USA). Gene mapper software v5.0 was used for alleles call and haplotypes were constructed manually.

## Results

### Clinical Exome Sequencing (CES) Analysis

Three affected families with ARNSHL were studied (**Figure 1A**). The proband of each affected family was screened for variants in the *GJB2* gene using PCR and Sanger Sequencing and no variant has been detected. Thus, we performed a CES analysis including 126 genes implicated in HL. The total numbers of variants were 327640 for YMN3, 314150 for YMN5, and 267190 for YMN8. As all families were consanguineous, we considered only homozygous variants located in the HL-related genes and with a frequency less than 0.01 (**Table 1**). Subsequently, VEP (Variant Effect Predictor) tool, LoFTool score, LRT pred, Mutation Taster pred and VarSome were used to predict the functional impact of these remaining variants. The LoFTool score identified only one variant in the *CLDN14* gene (c.414G>A) as damaging, LRT pred showed that the same variant is D (deleterious) and the Mutation Taster pred showed that it is a disease causing (D, D, D, D, D). VarSome also confirmed that this variant in the *CLDN14* gene is predicted to be pathogenic.

**Table 1:** Predicted impact of the remaining variants after filtration of the CES results.

Gene	Patient ID	Variant type	cDNA variant	Amino acid change	Classification
*BDP1*	YMN3	Exonic-NC*	n.2439A>T	*NA	Likely benign
*CLDN14*	YMN3 YMN5 YMN8	Nonsense	c.414G>A	p.Trp138Ter	Pathogenic
*SLC26A2*	YMN5	Missense	c.1721T>C	p.Ile574Thr	Benign
*NARS2*	YMN5	5UTR	c.-854C>T	*NA	Uncertain significance
*CDH23*	YMN8	5UTR	c.-35_-31dup	*NA	Likely benign

*NC, Exonic Non coding; *NA, Not available.

**Figure 2:**
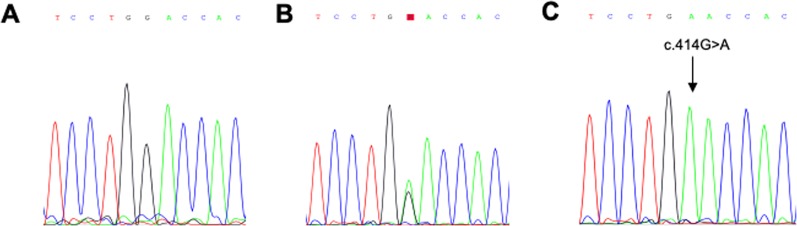
Electropherograms. Representative electropherogram of a normal homozygous individual **(A)**, a heterozygous individual **(B)** and an affected homozygous individual **(C)**.

To confirm these findings, we sequenced the 7th exon of the *CLDN14* using Sanger sequencing (**Figure 2**). The analysis revealed a co-segregation of the c.414G>A variant in the three families. This co-segregation was also confirmed by PCR-RFLP using the AvaII restriction enzyme (**Figure 1C**). In fact, all affected individuals were homozygous for this nucleotide transition, parents were heterozygous, and unaffected siblings were either heterozygous or homozygous normal. 

### Genotyping and Haplotype Analysis

Based on the previous results shown in **Table 1**, we assumed that the nonsense variant p.Trp138Ter (c.414G>A) is a common founder variant. To confirm our hypothesis, four short tandem repeats (STR) markers (D21S1252, D21S168, D21S267, D21S1894) were analyzed in the three affected families (**Figure 3A**). Apart from marker D21S1894, a homozygous haplotype for the alleles 246,104 and 188, of the markers D21S1252, D21S168, and D21S267, has been detected in all affected individuals, while parents and healthy individuals were heterozygous for this particular haplotype (**Figure 3B**).

## Discussion

In this study, we analyzed 3 Yemeni families with ARNSHL. As variants in the *GJB2* gene are among the most common variants in Middle East (Shahin et al., 2002; Tlili et al., 2017), known to be related to HL and is commonly used for clinical screening (Zheng et al., 2015), we first pre-screened the affected individuals for variants in this gene. Due to the absence of pathogenic variants within the *GJB2* gene, we further analyzed the affected samples by CES. The CES results allowed us to identify a novel nonsense variant p.Trp138Ter (c.414G>A) in the *CLDN14* gene. The co-segregation of this variant with the disease was confirmed by Sanger sequencing and PCR-RFLP. While most of the variants in *CLDN14* that lead to hearing loss in different populations were missense variants (**Supplementary Table 3**), nonsense variants were only found in this study and in one family from Pakistan (Lee et al., 2012). Variants of *CLDN14* have been previously associated with hearing loss in Canada, Pakistan, India and Morocco (Wilcox et al., 2001; Bashir et al., 2010; Lee et al., 2012; Bashir et al., 2013; Charif et al., 2013; Pandey et al., 2017; Pater et al., 2017) (**Supplementary Table 3**). In addition, some studies showed the absence of variants in the *CLDN14* gene for other populations such as Tunisia, Turkey and China (Uyguner et al., 2003; Belguith et al., 2009; Lu et al., 2018).

The new c.414G>A variant within the *CLDN14* gene showed a phenotypic variability among the 3 Yemeni families. In fact, the audiograms revealed that the affected members have moderate to profound congenital hearing loss (**Figure 1B**). The HL phenotypic variability observed in our study has been also reported in different families with *CLDN14* variants (Wilcox et al., 2001). A previous study that compared the audiograms of affected Pakistani individuals with *CLDN14* variants, showed that the studied patients presented moderate to severe, moderately severe to profound, and severe to profound HL. This phenotypic variability could be explained by both the nature of the pathogenic variant and the possibility of interaction with modifier genes (Bashir et al., 2010; Bashir et al., 2013). For the Yemeni families studied here, and as the variant is common, the phenotypic variability is probably due to modifier genes and/or environmental factors.

Tight junctions in the inner ear are important as they are essential to sustain the paracellular permeability between endolymph and the adjacent tissue and maintain the apical-basal polarity within the cell. Thus, tight junctions are considered a dynamic barrier between the external and internal environments (Gupta and Ryan, 2010). Variants occurring in tight junction genes such as claudin-14 (*CLDN14*) have been shown to be involved in HL (Nunes et al., 2012). Claudin 14 is highly expressed in the kidney, liver and inner ear (Wilcox et al., 2001; Ben-Yosef et al., 2003). In the inner ear, *CLDN14* plays a key role during the auditory process by recycling potassium ions from the hair cells back to the endolymph (Duman and Tekin, 2012). *In vitro* studies, mouse knockout models and human phenotype studies demonstrated that this gene is very crucial for a normal function of the auditory system (Wilcox et al., 2001; Ben-Yosef et al., 2003; Angelow et al., 2008). In fact, it has been shown that *Cldn14*-null mice exhibit HL due to the rapid deterioration and the loss of function of their cochlear outer hair cells (OHCs) shortly after birth (Ben-Yosef et al., 2003).

Since the c.414G>A variant was observed in three unrelated families, we genotyped four microsatellite markers to test its founder effect. It was preferable to use microsatellite markers rather than SNPs because they are more polymorphic and they provide efficient and informative genotyping as they are highly mutable often with 15 or more alleles in any populations (Baird et al., 2008). Our analysis confirmed the founder effect of the c.414G>A variant. This is the second documented evidence for a founder variant in the *CLDN14* gene. The first founder effect has been reported for the c.488C>T variant that was predominant in the island population of Newfoundland (Pater et al., 2017).

In sum, we report here the first variant responsible for hearing loss in Yemeni population. This variant is a nonsense variant located within the *CLDN14* gene and has a founder effect.

## Data Availability Statement

The datasets analyzed in this manuscript are not publicly available. Requests to access the datasets should be directed to atlili@sharjah.ac.ae. 

## Ethics Statement

The studies involving human participants were reviewed and approved by University of Sharjah Research Ethics Committee (No. REC-15-11-P004). The patients/participants provided their written informed consent to participate in this study.

## Author Contributions

AT designed the study. AT and AM supervised the study. WM performed the experiments and wrote the manuscript. AT, WM, and MM revised and edited the manuscript. AT, WM, MM, and SA analyzed the data. All authors have read and approved the manuscript.

## Funding

The work presented here was funded by the University of Sharjah and *Sheikh Hamdan Bin Rashid Al-Maktoum Award for Medical Sciences*.

## Conflict of Interest

The authors declare that the research was conducted in the absence of any commercial or financial relationships that could be construed as a potential conflict of interest.

## Abbreviations

NSHL, Non-syndromic Hearing Loss; ARNSHL, Autosomal Recessive Non-Syndromic Hearing Loss; HL, Hearing Loss; PCR, Polymerase Chain Reaction; RFLP, Restriction Fragment Length Polymorphism; *CLDN14*, Claudin 14; CES, Clinical exome sequencing.
