# Supplementary material for: A Novel Nonsense Mutation (c.414G>A; p.Trp138*) in CLDN14 Causes Hearing Loss in Yemeni Families: A Case Report
Source: Front Genet. 2019 Nov 8;10:1087. doi: 10.3389/fgene.2019.01087 (PMC6856671; doi:10.3389/fgene.2019.01087)
Supplement: Supplementary file 1 [file DataSheet_1.docx]

**Supplementary tables:**

**Table S1**. Summary of clinical data for hearing loss in the three families.

| **Family** | **Subject** | **Gender** | **Age  (y.o.)** | ***PTA (dB HL)** | **Hearing impairment** | **Audiogram Shape** | **Other symptoms** |
| --- | --- | --- | --- | --- | --- | --- | --- |
| YMN-I | YMN2 | Female | 25 | L:60 R:80 | Moderate to severe | Sloppy | No |
|  | YMN3 | Female | 23 | L:70 R:80 | Severe | Sloppy | No |
| YMN-II | YMN4 | Female | 29 | L:90 R:100 | Severe-profound | Sloppy | No |
|  | YMN5 | Female | 14 | L:70 R:80 | Severe | Sloppy | No |
|  | YMN6 | Female | 21 | L&R:80 | Severe | Sloppy | No |
| YMN-III | YMN8 | Female | 25 | L&R:100 | Profound | Sloppy | No |
|  | YMN9 | Female | 29 | L:90 R:100 | Severe to profound | Sloppy | No |

*PTA: Pure-tone air-conduction average

L: left ear, R: right ear

**Table S2.** Sequences of primers, melting temperature and expected PCR sizes

| **Assay** | **Primer name** | **Forward Sequence** | **Reverse Sequence** | **Tm (C)** | **Size (bp)** |
| --- | --- | --- | --- | --- | --- |
| **Genotyping** | D21S1252 | **^VIC^**TCTGTCTTTGTCTCACTATCTG | GCAATGCTCTGTGGCT | 55° | 247-251 |
|  | D21S267 | **^NED^**ATGGATCTGGATTTCTATCTTC | CCTCCAACCTGGGTGA | 55° | 175-203 |
|  | D21S1894 | **^PET^**ATGAGGCTCAATGCTATTGGAGTGC | AAAGCCAGCTACAAGTCTTGCTGC | 55° | 169-201 |
|  | D21S168 | **^FAM^**ATGCAATGTTATGTAGGCTG | CGGCATCACAGTCTGATAAA | 55° | 104-118 |
| **Sequencing** | CLDN14-seq | ACCACCATCCTGCCGCACTG | TGTTTGCAGTGGTCGTGGTG | 55° | 550 bp |
| **PCR-RFLP** | CLDN14 | CATTTCCTTTCTCTCCCTGCT | GACATTTCCTCGCATTCACA | 55° | 987 bp |

**Table S3.** Reported *CLDN14 v*ariants in different populations.

| **Population** | **Mutation** | **Protein** | **Variant** | **Ref** |
| --- | --- | --- | --- | --- |
| Greece and Spanish mix | c.301G>A | p.G101R | missense | (Wattenhofer et al., 2005) |
| India | c.254T>A | p.Val85Asp | missense | (Pandey et al., 2017) |
| Morocco | c.11C>T | p.T4M | missense | (Charif et al., 2013) |
| Newfoundland | c.488C>T | p.A163V | missense | (Pater et al., 2017) |
| Pakistan | c.254T>A | p.Val85Asp | missense | (Bashir et al., 2010) |
|  | c.259-260TC>AT c.281C>T c.242G>A c.254T>A c.398delT | p.Ser87Ile p.Ala94Val p.Arg81His p.Val85Asp p.Met133ArgfsX23 | missense missense missense missense deletion | (Bashir et al., 2013) |
|  | c.254T>A c.398delT | p.Val85Asp p.Met133Argfsx23 | missense deletion | (Wilcox et al., 2001) |
|  | c.167G>A c.242G>A 694G>A c.254T>A | p.w56* p.Arg81His p.Gly232Arg p.Val85Asp | nonsense missense missense missense | (Lee et al., 2012) |
| Yemen | c.414G>A | p.Trp138Ter | nonsense | current study |
