# Supplementary figures and images for: A Novel Nonsense Mutation (c.414G>A; p.Trp138*) in CLDN14 Causes Hearing Loss in Yemeni Families: A Case Report
Source: Front Genet. 2019 Nov 8;10:1087. doi: 10.3389/fgene.2019.01087 (PMC6856671; doi:10.3389/fgene.2019.01087)

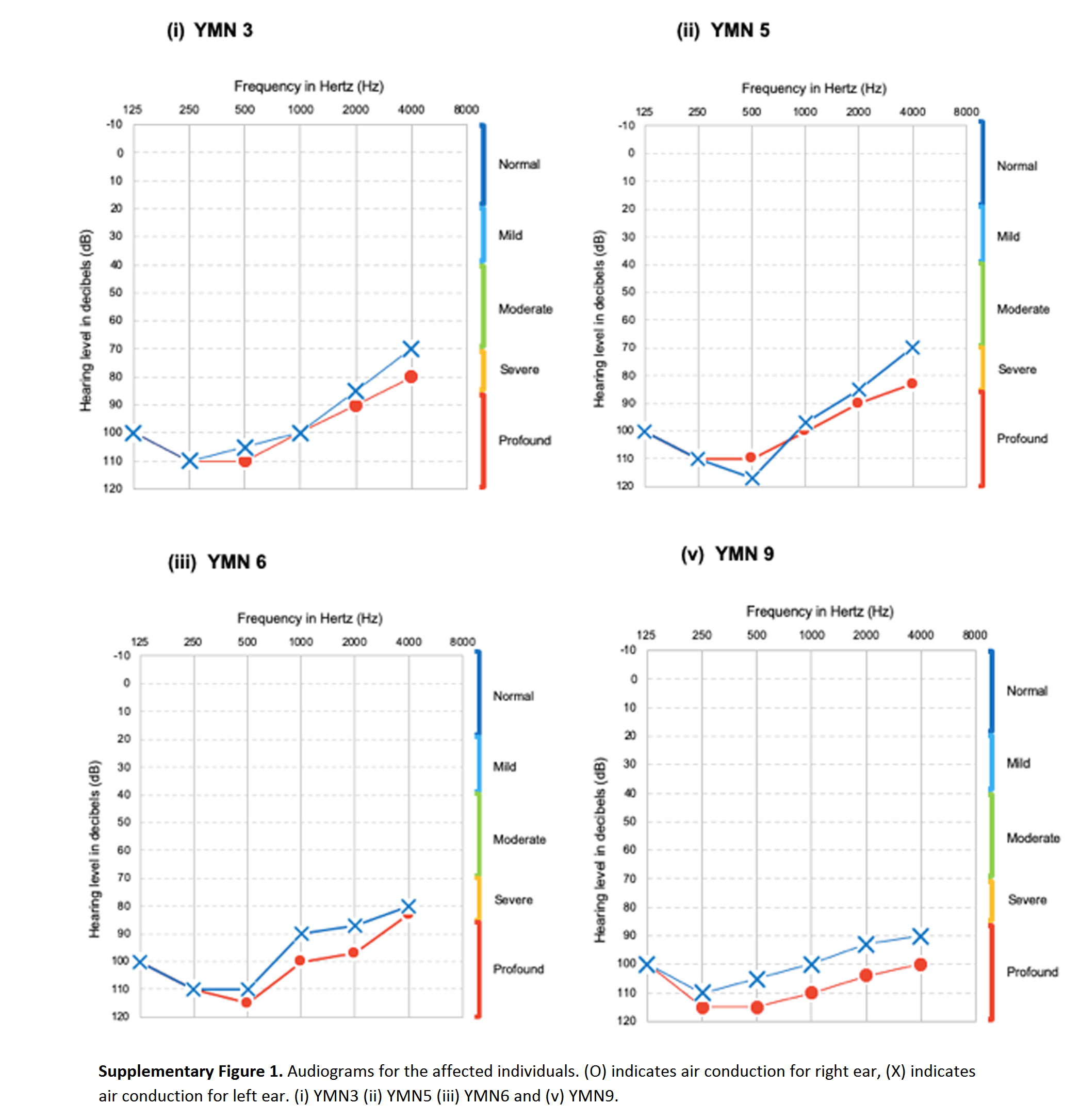

Supplement: Supplementary file 2 [file Image_1.tif]
